# Patient-level costs of central line-associated bloodstream infections caused by multidrug-resistant microorganisms in a public intensive care unit in Brazil: a retrospective cohort study

**DOI:** 10.5935/0103-507X.20220313-en

**Published:** 2022

**Authors:** Antonio Paulo Nassar Júnior, Isabella Lott Bezerra, Daniel Tavares Malheiro, Maria Dolores Montoya Diaz, Guilherme Paula Pinto Schettino, Adriano José Pereira

**Affiliations:** 1 IMPACTO-MR Program, Health Economics, Hospital Israelita Albert Einstein - São Paulo (SP), Brazil.; 2 Economics Department, Faculdade de Economia, Administração, Contabilidade e Atuária, Universidade de São Paulo - São Paulo (SP), Brazil.; 3 Social Responsibility Institute, Hospital Israelita Albert Einstein - São Paulo (SP), Brazil.

**TO THE EDITOR,**

Hospital-acquired infections (HAIs) are a major threat to patients and health care systems.^(1)^ Hospital-acquired infections are associated with increased mortality and prolonged hospital length of stay.^(2)^ However, it is not clear whether HAIs caused by multidrug-resistant (MDR) pathogens acquired in intensive care units (ICUs) are associated with increased costs when compared to HAIs caused by susceptible pathogens.^(3)^

Central line-associated bloodstream infections (CLABSIs) are among the most common HAIs in ICUs.^(4)^ Central line-associated bloodstream infections are also associated with increased costs, but it is not clear whether CLABSIs caused by MDR pathogens impose additional costs than those already imposed by infections caused by susceptible pathogens.^(5)^ Therefore, this study aimed to assess the economic burden of MDR CLABSI in an ICU in a public hospital in Brazil.

We carried out a retrospective cohort study carried out in the ICU of a tertiary public hospital located in the city of São Paulo, Brazil. The local and municipal Institutional Review Board approved the study (CAAE 20732619.6.0000.0071 and CAAE 20732619.6.3001.0086).

We included all patients aged 18 years or older admitted to the ICU who used a central-venous catheter during their ICU stay from January 1st, 2016, to December 31st, 2020. We excluded patients who were admitted for solid-organ transplants because surgery for transplantation per se was performed in another hospital. We also excluded patients admitted due to pregnancy, childbirth, and puerperium.

We categorized patients as those with multidrug-resistant CLABSI (MDR-CLABSI) and non-multidrug-resistant CRBMI (nMDR-CLABSI). The following bacteria and fungi were defined as MDR microorganisms: *Acinetobacter baumannii* and *Pseudomonas aeruginosa* resistant to carbapenems and/or polymyxins; *Enterobacteriaceae* resistant to third and fourth generation cephalosporins, carbapenems, and/or polymyxins; *Enterococcus faecium* resistant to vancomycin; *Staphylococcus aureu*s resistant to methicillin; coagulase-negative *Staphylococcus* resistant to methicillin; and *Candida* species resistant to imidazoles.

The absorption costing method was applied with a top-down approach.^(6)^ The total cost of a hospital stay is the sum of five cost categories: fixed costs (activities performed by clinicians, water, and energy costs), laboratory and imaging, medical material, drugs, and procedures. We calculated all costs considering the current costs in the unit in February 2021. We converted costs in US dollars considering the mean exchange rate in February 2021 (1 USD = BRL 5.4159).

The primary outcome was the total hospital cost per patient. Secondary outcomes were fixed, variable, and category daily costs. Tertiary outcomes were hospital mortality, ICU length of stay (LOS), and hospital LOS.

All categorical data are presented as absolute numbers and percentages and compared with chi-square or Fisher’s test, as appropriate. All continuous data are presented as medians and interquartile ranges and compared with the Mann-Whitney test.

The main comparison was made between patients with MDR-CLABSI and nMDR-CLABSI. Second, we used a propensity score matching method to compare patients with MDR-CLABSI and patients without CLABSI and to compare patients with nMDR-CLABSI with patients without CLABSI. The propensity score was calculated by fitting two logistic regression models. The dependent variable of the logistic regressions was the occurrence of a CLABSI, and the independent variables (confounders) were age, sex, diagnosis at admission, Charlson comorbidity index, and Simplified Acute Physiology Score 3 (SAPS 3). We used the nearest neighbor matching method to match patients with CLABSI to patients without CLABSI. Each patient with a CLABSI was matched with 10 patients without CLABSIs.

**Figure 1:**
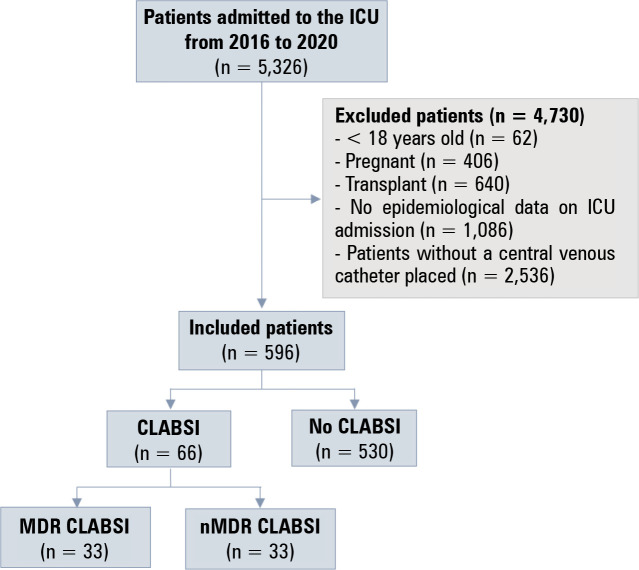
Study flowchart.

A total of 5,326 patients were admitted to the ICU during the study period, and 596 (11.2%) patients used a central venous catheter (Figure 1). A total of 66 (11.1%) had a CLABSI. Thirty-three patients had MDR-CLABSI, and 33 patients had nMDR-CLABSI.

ICU - intensive care unit; CLABSI - central line-associated bloodstream infection; MDR - multidrug-resistant; nMDR - non-multidrug resistant

Patients with and without CLABSI were not different regarding age, sex, comorbidities, type, and severity at admission. The most common reason for admissions was infectious diseases. However, patients with MDR-CLABSI had more admissions due to respiratory and genitourinary diseases and fewer admissions due to circulatory diseases. Patients with CLABSIs had longer ICU and hospital LOS. Patients with MDR-CLABSI had higher hospital mortality than patients with nMDR-CLABSI and patients without CLABSI in the unmatched cohort (Table 1). The etiologic agents of the CLABSI are shown in table 2.

**Table 1:** Baseline characteristics and outcomes of included patients before propensity score matching

	MDR-CLABSI (n = 33)	nMDR-CLABSI (n = 33)	Without CLABSI (n = 530)	p value
Age (years)	61 (45 - 67.5)	61(48 - 68)	59 (47 - 69)	0.89
Sex				0.30
Female	15 (45.5)	10 (30.3)	233 (44.0)	
Male	18 (54.5)	23 (69.7)	297 (56.0)	
Charlson comorbidity index	1 (1 - 3)	2 (1 - 3.5)	2 (1 - 3)	0.46
SAPS 3	47 (41.5 - 54.5)	47 (41.5 - 53.0)	48 (50 - 56)	0.96
Type of admission				0.83
Medical	1 (3.0)	2 (6.1)	507 (95.7)	
Surgical	32 (97.0)	31 (93.9)	23 (4.3)	
Reason for admission				< 0.01
Infectious and parasitic diseases	16 (48.5)	16 (48.5)	262 (49.4)	
Diseases of the respiratory system	4 (12.1)	1 (3.0)	51 (9.6)	
Diseases of the genitourinary system	3 (9.1)	2 (6.1)	28 (5.3)	
Diseases of the circulatory system	2 (6.1)	11 (33.3)	125 (23.6)	
Diseases of the nervous system	0 (0)	2 (6.1)	12 (2.3)	
Neoplasms	1 (3.0)	1 (3.0)	12 (2.3)	
Injury, poisoning and certain other consequences of external causes	2 (6.1)	0 (0)	14 (2.6)	
ICU LOS (days)	25 (12.5 - 32.5)	23 (12.5 - 42.0)	7 (3 - 14)	< 0.01
Hospital LOS (days)	33 (26 - 56)	48 (33.5 - 78)	14 (8 - 25)	< 0.01
Hospital mortality	19 (57.6)	10 (30.3)	149 (28.1)	< 0.01

MDR - multidrug resistant; CLABSI - central line associated bloodstream infection; nMDR - non-multidrug resistant; SAPS 3 - Simplified Acute Physiology Score 3; ICU - intensive care unit; LOS - length of stay. The results are expressed as the median (interquartile range) or n (%).

**Table 2:** Etiologic pathogens of central line-associated bloodstream infections

	Patients (n)
Multidrug-resistant infections	
Pathogen	
Coagulase-negative *Staphylococcus*	12
*Klebsiella pneumoniae*	7
*Staphylococcus aureus*	4
*Enterococcus faecium*	4
*Serratia marcenses*	2
*Candida spp*	2
*Acinetobacter spp*	1
*Pseudomonas aeruginosa*	1
*Escherichia coli*	1
Non-multidrug-resistant infections	
Pathogen	
*Candida spp*	10
*Enterococcus faecalis*	6
*Klebsiella pneumoniae*	6
*Serratia marcescens*	4
*Klebsiella pneumoniae*	3
*Pseudomonas aeruginosa*	2
*Enterobacter aerogenes*	1
*Escherichia coli*	1
*Citrobacter freundii*	1

When compared to propensity-matched patients without CLABSI, patients with nMDR-CLABSI had longer ICU LOS but not hospital LOS or hospital mortality. On the other hand, patients with MDR-CLABSI had longer ICU and hospital LOS and a higher hospital mortality rate than propensity-matched patients without CLABSI (Table 3).

**Table 3:** Clinical characteristics and outcomes of patients with catheter-related bloodstream infection and propensity-matched patients without catheter-related bloodstream infection

	MDR-CLABSI (n = 33)	Without CLABSI (n = 330)	p value	nMDR-CLABSI (n = 33)	Without CLABSI (n = 330)	p value
Age (years)	61 (45 - 67.5)	61 (50.75 - 71.0)	0.25	61(48 - 68)	60 (48 - 70)	0.74
Sex			0.51			0.93
Female	15 (45.5)	170 (51.5)		10 (30.3)	106 (32.2)	
Male	18 (54.5)	160 (48.5)		23 (69.7)	224 (67.8)	
Charlson comorbidity index	1 (1 - 3)	1 (0 - 3)	0.73	2 (1 - 3.5)	2 (1 - 3.5)	0.89
SAPS 3	47 (41.5 - 54.5)	50 (42 - 59)	0.46	47 (41.5 - 53.0)	49 (42 - 55.5)	0.50
Reason for admission			< 0.01			0.02
Infectious and parasitic diseases	16 (48.5)	195 (59.1)		16 (48.5)	119 (59.6)	
Diseases of the respiratory system	4 (12.1)	51 (15.5)		1 (3.0)	0 (0)	
Diseases of the genitourinary system	3 (9.1)	22 (6.7)		2 (6.1)	6 (1.8)	
Diseases of the circulatory system	2 (6.1)	21 (6.4)		11 (33.3)	104 (31.6)	
Neoplasms	2 (6.1)	25 (7.6)		1 (3.0)	11 (3.3)	
Diseases of the nervous system	2 (6.1)	14 (4.2)		2 (6.1)	12 (3.6)	
ICU LOS (days)	25 (12.5 - 32.5)	7 (3 - 14)	< 0.01	23 (12.5 - 42.0)	8 (4 - 16.5)	< 0.01
Hospital LOS (days)	33 (26 - 56)	13 (7 - 24)	< 0.01	48 (33.5 - 78)	49 (42 - 55.5)	0.50
Hospital mortality	19 (57.6)	125 (37.9)	0.03	10 (30.3)	90 (27.4)	0.72
Total Hospital Cost (in USD)	33,808.92 (24,554.20 - 46,555.88)	10,189.69 (5,583.13 - 19,132.20)	< 0.01	30,814.39 (23,600.30 - 62.951,80)	10,580.27 (5,634.85 - 19,102.36)	< 0.01

MDR - multidrug resistant; CLABSI - central line associated bloodstream infection; nMDR - non-multidrug resistant; SAPS 3 - Simplified Acute Physiology Score 3; ICU - intensive care unit; LOS - length of stay. The results are expressed as the median (interquartile range) or n (%).

Patients with MDR-CLABSI have increased hospital costs compared with propensity-score matched patients without CLABSI [$33,808.92 ($24,554.20 - $46,555.88) *versus* $10,189.69 (5,583.13 - 19,132.20); p < 0.01] (Table 3). Patients with nMDR-CLABSI also had higher total hospital costs than propensity-score matched patients without CLABSI [$30,814.39 ($23,600.30 - $62.951,80) *versus* $10,580.27 (5,634.85 - 19,102.36); p < 0.01] (Table 3).

There were no differences between patients with MDR-CLABSI and nMDR-CLABSI on total hospital costs [$33,808.92 ($24,554.20 - $46,555.88) *versus* $30,814.39 ($23,600.30 - $62.951,80); p = 0.99]. There were also no differences in total fixed and variable costs (laboratory and imaging, medical material, drugs, and procedures costs) (Table 4). Daily total and daily fixed costs were also not different between patients with MDR-CLABSI and nMDR-CLBSI. However, patients with MDR-CLABSI had increased variable daily costs compared to patients with nMDR-CLABSI [$397.73 ($251.12 - $717.18) *versus* $291.42 ($128.12 - $526.37); p = 0.04]. This difference was mainly explained by higher costs of medical materials and procedures among patients with MDR-CLABSI.

**Table 4:** Categories of direct cost of central line-associated bloodstream infection

Category of cost	Total cost			Daily cost		
	MDR CLABSI (US$)	nMDR CLABSI (US$)	p value	MDR CLABSI (US$)	nMDR CLABSI (US$)	p value
Fixed costs	20,373.89 (13,109.97 - 25,014.58)	19,484.20 (13,883.27 - 33,599.38)	0.54	1039.13 (754.53 - 1,919.41)	811.84 (447.99 - 1,449.20)	0.19
Variable costs	14,223.96 (10,030.57 - 21,488.22)	11,814.26 (8,245.82 - 20,935.18)	0.57	397.73 (251.12 - 717.18)	291.42 (128.12 - 526.37)	0.04
Laboratory and imaging	2,024.79 (1,565.73 - 3,536.86)	2,465.72 (1,583.53 - 3,483.28)	0.52	125.06 (73.45 - 252.63)	91.18 (53.16 - 173.38)	0.15
Medical material	2,315.44 (1,815.56 - 3,324.57)	1,871.35 (1,436.08 - 3,669.59)	0.28	142.52 (87.43 - 222.44)	78.69 (35.35 - 193.76)	0.02
Drugs	4,650.60 (1,895.05 - 7,051.40)	4,013.25 (2,319.35 - 6,007.50)	0.78	176.97 (126.71 - 347.35)	159.32 (67.36 - 255.92)	0.09
Procedures	4,495.04 (3,585.09 - 7,721.94)	4,549.98 (1,740.49 - 7,969.25)	0.37	281.48 (188.11 - 404.46)	168.52 (84.86 - 336.35)	0.02
Total costs	33,888.92 (24,554.20 - 46,555.88)	30,814.39 (23,600.30 - 62,951.80)	0.99	1,980.31 (1,177.17 - 3,503.02)	1,287.11 (625.60 - 2,395.22)	0.10

MDR - multidrug resistant; CLABSI - central line associated bloodstream infection; nMDR - non-multidrug resistant. The results expressed as median (interquartile range).

Thus, CLABSIs caused by MDR pathogens were not associated with increased ICU hospital charges when compared to CLABSIs caused by nMDR pathogens in this cohort. Nevertheless, they were associated with increased consumption of medical materials and procedures and higher hospital mortality. Both CLABSIs caused by MDR and nMDR pathogens were associated with an increase of approximately three times in hospital charges. However, this was a small, single-center study with a slightly higher incidence of CLABSI than similar studies.^(7)^ The small sample size also may not have had sufficient power to detect small cost differences. Additionally, we cannot exclude the possibility that there was some selection bias since we could not retrieve data from 1,086 patients. Larger studies evaluating direct costs should assess whether MDR infections are more costly, especially to public health care systems, and whether intervention measures that may decrease HAIs are cost-effective in these settings.
